# Vitamin D Supplementation for Children with Epilepsy on Antiseizure Medications: A Randomized Controlled Trial

**DOI:** 10.3390/children11101187

**Published:** 2024-09-28

**Authors:** Fahad A. Bashiri, Abrar Hudairi, Muddathir H. Hamad, Lujain K. Al-Sulimani, Doua Al Homyani, Dimah Al Saqabi, Amal Y. Kentab, Reem A. Al Khalifah

**Affiliations:** 1 Department of Pediatrics, College of Medicine, King Saud University, Riyadh 11461, Saudi Arabia; ahudairi@ksu.edu.sa (A.H.); mudhamad@ksu.edu.sa (M.H.H.); akentab@ksu.edu.sa (A.Y.K.); ralkhalifah@ksu.edu.sa (R.A.A.K.); 2 Division of Pediatric Neurology, Department of Pediatrics, College of Medicine, King Saud University Medical City, King Saud University, Riyadh 11461, Saudi Arabia; 3 Division of Pediatric Endocrinology, Department of Pediatrics, College of Medicine, King Saud University Medical City, King Saud University, Riyadh 11461, Saudi Arabia; lalsulimani@moh.gov.sa (L.K.A.-S.); dalhemayani@moh.gov.sa (D.A.H.); 4 College of Medicine Research Center, King Saud University, Riyadh 11461, Saudi Arabia; dalsaqabi@ksu.edu.sa (D.A.S.)

**Keywords:** vitamin D supplementation, vitamin D deficiency, epilepsy, children, antiseizure medications, Saudi Arabia

## Abstract

Background: Antiseizure medications (ASMs) are crucial for managing epilepsy in children. However, a well-documented side effect of ASMs is their impact on bone health, often due to interference with vitamin D metabolism. This can lead to vitamin D deficiency in children with epilepsy. This study aimed to determine if a daily dose of 400 IU or 1000 IU would maintain adequate vitamin D levels in children with epilepsy. Methods: A phase IV randomized controlled trial enrolled children aged 2–16 years with epilepsy and receiving antiseizure medications. Children were divided into two groups: the monotherapy group, which was defined as children on one antiseizure medication (ASM), and the polytherapy group, which was defined as children receiving two or more ASMs. Eligible children with levels above 75 nmol/L were randomized to receive a maintenance dose of either 400 IU/day or 1000 IU/day of cholecalciferol. Baseline and 6-month assessments included demographic data, anthropometric measurements, seizure type, medications, seizure control, and 25(OH)D level. Results: Out of 163 children, 90 were on monotherapy and 25 on polytherapy. After 6 months of vitamin D maintenance, the proportion of children with 25(OH)D concentration below 75 nmol/L was 75.0% in the 400 IU group and 54.8% in the 1000 IU group. In the monotherapy group, baseline seizure-free children increased from 69% to 83.6% after treating vitamin D deficiency. Conclusion: Daily vitamin D supplementation with 1000 IU may be beneficial for children with epilepsy, particularly those receiving monotherapy, to maintain sufficiency and potentially improve seizure control.

## 1. Introduction

Vitamin D deficiency is prevalent worldwide across all age groups. In Saudi Arabia, about 40.6% to 97.8% of children between 6 and 15 years old are affected by vitamin D insufficiency and deficiency [1]. Vitamin D plays a critical role in maintaining bone health, muscle strength, immune function, cellular growth and differentiation, neurotransmission, and immune response in the central nervous system [2]. Vitamin D is essential for neuronal function and neuroprotection, and the deficiency has been linked to many neurological disorders [3]. Additionally, it is proposed that low vitamin D levels tend to increase seizure frequency in children with epilepsy. This observation was noticed in studies that investigated the link between seasonal variation and epilepsy. They found a significant fluctuation in seizure occurrence throughout the year, with the lowest number of seizures in summer and the highest in winter [4,5,6]. This increase in seizure frequency during winter was linked to diminished vitamin D levels.

Vitamin D metabolism begins with the synthesis of vitamin D3 in the skin from UVB exposure or through dietary intake. Both vitamin D3 (cholecalciferol) and D2 (ergocalciferol) are hydroxylated in the liver by 25-hydroxylase to form 25-hydroxyvitamin D [25(OH)D]. The second hydroxylation occurs in the kidneys, where 25(OH)D is converted by the enzyme 1α-hydroxylase into the biologically active form, 1,25-dihydroxyvitamin D [1,25(OH)2D] [7]. This active form binds to the vitamin D receptor (VDR) in various tissues, regulating calcium and phosphate homeostasis, promoting bone health, and modulating immune function. Vitamin D metabolism can be disrupted by certain antiseizure medications (ASMs), particularly those that induce the hepatic cytochrome P450 enzyme system, leading to increased catabolism of vitamin D and its metabolites. This enzyme induction accelerates the conversion of 25-hydroxyvitamin D [25(OH)D] into inactive forms, thereby reducing the availability of the active form, 1,25-dihydroxyvitamin D [1,25(OH)2D], which is crucial for calcium homeostasis and bone health.

Antiseizure medications (ASMs) can have a detrimental effect on bone health by compromising bone quality and increasing the likelihood of fractures [8]. This is thought to occur due to the interaction between ASMs and vitamin D metabolism, decreased osteoblast proliferation, and changes in collagen synthesis. Research involving cohorts has indicated that enzyme-inducer ASMs tend to lower the concentration of 25 hydroxyvitamin D (25(OH)D), putting individuals with epilepsy at a significantly higher risk of vitamin D deficiency compared to those not on ASMs [9]. Studies have demonstrated that children with epilepsy, particularly those who are obese and taking enzyme-inducing ASMs, face an elevated risk of vitamin D deficiency [10,11,12,13,14,15,16,17,18,19,20,21,22]. However, there are variations in findings regarding the vitamin D-lowering impact of these same ASMs across different studies. This discrepancy could be attributed to differences in study design, geographic location, or dietary patterns among the study populations.

Two pilot studies investigated the impact of vitamin D treatment on adults with epilepsy and vitamin D deficiency. They observed a 30% reduction in the average seizure frequency compared to the control group [23,24]. To date, only one randomized controlled trial (RCT) has assessed the effect of providing 78 children aged 10–18 years who are on long-term ASM therapy with either 400 IU/day or 2000 IU/day of vitamin D over a period of one year, irrespective of their initial vitamin D levels [25]. The study reported similar bone mineral density (BMD) levels between both groups and comparable mean 25(OH)D concentrations after one year. However, it did not report seizure frequency.

Various scientific organizations have put forth recommendations for vitamin D supplementation in children. They typically recommend a daily maintenance dosage of 400 IU for all children. However, when it comes to children with epilepsy, there is a lack of conclusive evidence to recommend a specific maintenance dose. The American Academy of Pediatrics recommends 400 IU daily for healthy children and 400 to 1000 IU for those children who have recently undergone treatment for vitamin D deficiency. In contrast, the Endocrine Society 2011 guideline proposes a higher range of 600 to 1000 IU daily for children who are at an increased risk of developing vitamin D deficiency [26,27]. Therefore, we aim to determine whether a daily supplementation of 400 IU, as a standard dose for healthy children, or 1000 IU, recommended for children with an increased risk of vitamin D deficiency over 6 months of treatment, is effective in maintaining optimal levels of 25(OH)D in children with epilepsy who have a normal 25(OH)D concentration at baseline.

## 2. Methods

The design of this study has been described previously (NCT03536845) [28]. Briefly, we performed a phase IV pragmatic randomized controlled open-label trial. We recruited children aged 2–16 years diagnosed with epilepsy and treated with ASMs attending the outpatient pediatric neurology clinic at King Saud University Medical City, King Saud University in Riyadh, Kingdom of Saudi Arabia, from December 2017 to March 2021. Children were excluded if they have a pre-existing vitamin D metabolism problem such as vitamin D dependent rickets, malabsorption syndromes like celiac disease, inflammatory bowel disease, renal disease, hepatic disease, or children who are not safe to start vitamin D supplementation such as total corrected calcium > 2.5 mmol/L, 25(OH)D concentration > 250 nmol/L, or urine calcium: creatinine ration > 1.2 mol/mol, or >0.41 g/g. The trial was approved by the institutional review board at King Saud University (IRB no. E-17 2425). All study procedures complied with the Good Clinical Practice and Declaration of Helsinki. Informed consent and assent were obtained from the children and their parents.

According to family preference, children with 25(OH)D concentration < 75 nmol/L were treated with cholecalciferol 5000 IU/day or 35,000 IU once per week for 8 weeks and 30–75 mg/kg per day of elemental calcium in 3 divided doses for 4 weeks. Afterward, the 25(OH)D concentration was measured after completing the 8-week treatment course [26,27]. The treatment course was repeated until 25(OH)D concentration normalization was achieved.

Eligible children with 25(OH)D concentrations above 75 nmol/L were randomized to receive a maintenance dose of either 400 IU/day or 1000 IU/day of cholecalciferol (Novartis Vi-De 3 10 mL drops, 100 IU/drop) for 6 months. Children were asked not to consume other forms of vitamin D and multivitamins. Children were divided into two groups: the monotherapy group, which was defined as children on one antiseizure medication (ASM), and the polytherapy group, which was defined as children receiving two or more ASMs. The monotherapy group randomization was stratified blocked based on the type of ASM (enzyme inducer and non-enzyme inducer) and body mass index (BMI) of the child (<85th percentile or >85th percentile for age and sex). The P450 enzyme-inducer ASMs are carbamazepine, phenobarbital, and phenytoin. The non-enzyme-inducer ASMs are lamotrigine, levetiracetam, and valproate. Topiramate is considered an enzyme-inducer in higher doses [29]. At the same time, the polytherapy group randomization was stratified based on BMI only. The randomization schedule was produced by the online software, Sealed Envelope^TM^ (https://www.sealedenvelope.com (accessed on 2 December 2017)). The sequence allocation was concealed using opaque sealed envelopes drawn sequentially. The sequence generation and concealment were performed by a member of the study team who is not directly involved with patients.

### 2.1. Study Measures

To assess patient eligibility, safety before randomization, and risk factors for vitamin D deficiency, at baseline, we obtained demographic data, anthropometric measurement, the type of epilepsy (using the 2017 International League Against Epilepsy classification), ASMs with dosage, drug level, seizure control, laboratory workup: 25(OH)D concentration, ASM level, alkaline phosphatase, corrected calcium, parathyroid hormone, urine calcium: creatinine ratio, liver enzymes, renal function test.

After 3 months of randomization, children were invited to perform laboratory assessments of 25(OH)D concentration, ASM level (if applicable), alkaline phosphatase, corrected calcium, parathyroid hormone, and urine calcium: creatinine ratio. After 6 months of randomization, children were assessed in the clinic for anthropometric measurements, seizure control, ASM dosage, drug level, laboratory workup: 25(OH)D concentration, ASM level, alkaline phosphatase, corrected calcium, parathyroid hormone, and urine calcium: creatinine ratio.

### 2.2. Outcomes

Our primary outcome is the proportion of children with vitamin D insufficiency after 6 months of randomization. The insufficiency is defined as 25(OH)D concentration < 75 nmol/L, while deficiency is defined as 25(OH)D concentration < 50 nmol/L according to the Endocrine Society 2011 guidelines and the Canadian Pediatric Society 2007 guidelines [26,27,30]. These values are based on studies linking low vitamin D levels to bone health outcomes, including rickets in children and osteomalacia in adults. However, it is important to acknowledge the scientific debate over the optimal levels of vitamin D, particularly in relation to non-skeletal health outcomes. The criteria for deficiency and insufficiency are grounded in evidence, though they are not immune to the influence of evolving scientific understanding and, to some extent, commercial interests in vitamin D supplementation. Despite these factors, the chosen cut-offs represent a consensus aimed at reducing the risk of adverse skeletal outcomes, which remains a primary concern in clinical practice.

The secondary outcomes included the 25(OH)D concentration, the proportion of children with ASM treatment failure, and mean seizure frequency after 6 months of vitamin D supplementation. Treatment failure is defined as a composite outcome of a clinically significant increase in seizure frequency, a need to add new ASM to control seizure, or an increase in the ASM dose to control seizure that is not secondary to poor compliance, decreased ASM level, or intercurrent illness. Any change in seizure frequency by 50% from baseline is considered clinically significant. Children were considered seizure-free following a six-month period without experiencing any seizures. Additionally, for safety, we evaluated the proportion of children with hypercalcemia with total calcium > 0.7 mg/dL, 25 OH vit D level > 250 nmol/L, and urine calcium: creatinine ration > 1.2 mol/mol, or >0.41 g/g.

### 2.3. Statistical Analyses

We presented continuous, normally distributed data by mean and standard deviation (SD) and dichotomous data by N (%) data. The primary outcome was analyzed using intention-to-treat analysis (ITT) on the basis of the last observation carried forward. Within groups, proportion and rate comparisons were analyzed using the Chi-square test. Continuous outcomes were analyzed using an independent student *t*-test. We performed univariate linear regression analyses to evaluate the impact of vitamin D dose, BMI status, and enzyme inducer ASM on 25(OH)D concentration and duration of therapy. The analysis was performed using STATA version 16SE for Mac.

## 3. Results

**Figure 1:**
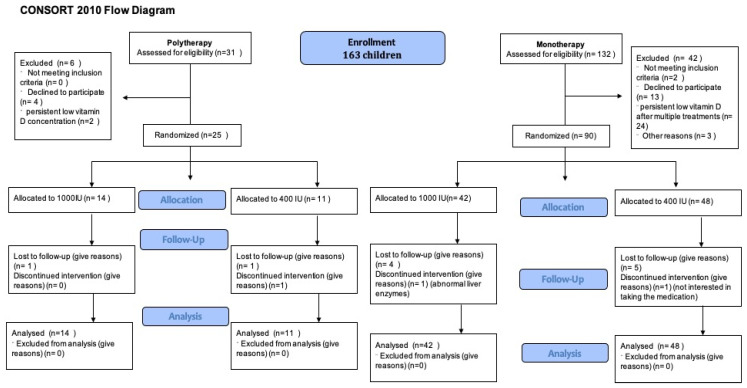
Patient flow diagram.

A total of 163 children were enrolled. Of them, 132 children were assessed on monotherapy and 31 children on polytherapy for eligibility. Following the eligibility assessment (see Figure 1), 90 children on monotherapy and 25 on polytherapy were randomized. Notably, 41 children (35.6%) were receiving enzyme inducer therapy. During the COVID-19 epidemic, we could only recruit 5 patients and randomize 20 patients [31]. The mean age of children was 9.1 ± 3.5 years; 85 (53.46%) were male (Table 1). At baseline, all groups had similar anthropometric measures and seizure patterns. Only 41 (25.79%) had 25(OH)D concentration > 75 nmol/L. All other children need to receive a vitamin D treatment course before randomization; 52 (53.61%) needed more than one treatment course over a median time of 5.6 (3.4, 9.9) months (see Table 1). There was no difference between children taking monotherapy or polytherapy in terms of needing multiple vitamin D treatment courses or duration of treatment courses. During the initial screening visit, 68 out of 98 children (69%) were seizure-free. After receiving treatment for vitamin D deficiency and before being randomly assigned to treatment groups, the number of seizure-free children increased to 82 out of 98 (83.6%). Risk difference 0.38 (95%CI 0.13, 0.63), the number needed to treat (NNT) is 2.6 children with vitamin D deficiency to become seizure-free, *p*-value 0.01. The difference was seen mostly in the monotherapy group, while no difference was observed in the polytherapy group.

**Figure 2:**
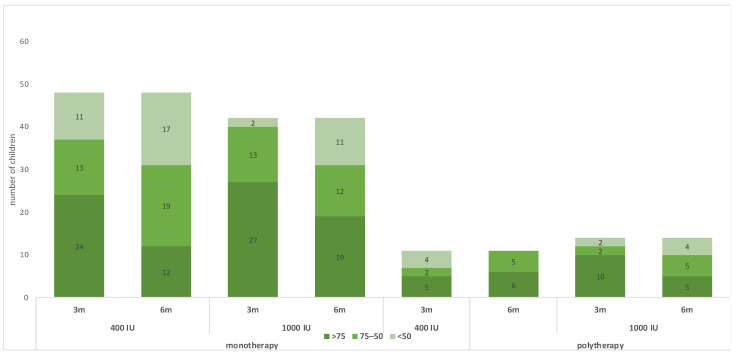
Changes in the 25(OH)D status during the trial.

**Figure 3:**
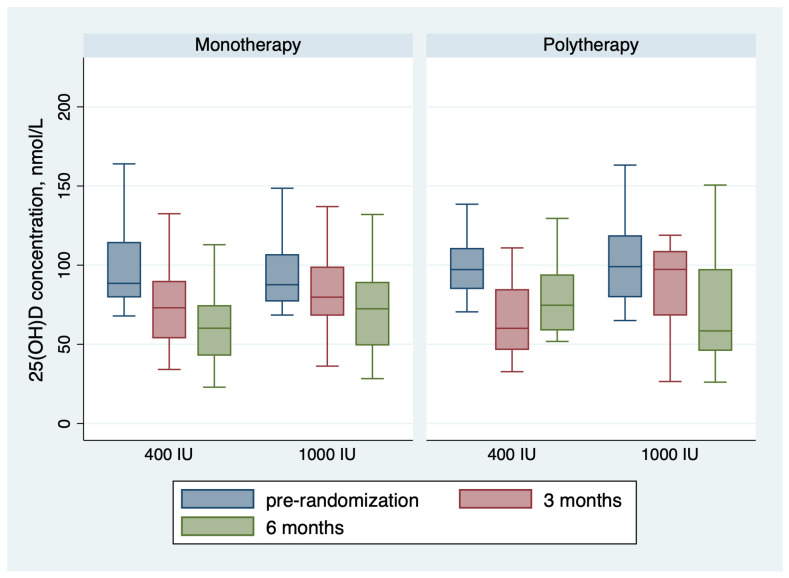
Changes in the 25(OH)D concentration during the trial.

Table 2 shows the outcomes of the trial. For the monotherapy group, after 6 months of receiving vitamin D maintenance, the proportion of children with 25(OH)D concentration < 75 nmol/L was 36 (75.0%) in the group receiving 400 IU and 23 (54.8%) in the group receiving 1000 IU; the risk difference with 1000 IU = −20% (95 CI% −0.40, −0.01), NNT = 5 patients, *p*-value = 0.044. The mean 25(OH)D concentration in the monotherapy group was 61.35 ± 22.45 nmol/L for children receiving 400 IU daily and 73.09 ± 29.03 for children receiving 1000 IU daily, with a *p*-value of 0.033 (Table 2 and Figure 2). The change in 25(OH)D concentration from randomization till 6 months was −38.13 ± 31.26 in the 400 IU group and −30.54 ± 45.28 in the 1000 IU (*p*-value 0.38) (Figure 2 and Figure 3). For the polytherapy group, the proportion of children with 25(OH)D concentration < 75 nmol/L was 5 (45.5%) in the group receiving 400 IU and 9 (64.3%) in the group receiving 1000 IU, the risk difference −20% (95 CI% −0.20, 0.58), *p*-value = 0.34. The mean 25(OH)D concentration was 78.79 ± 23.33 nmol/l for children receiving 400 IU daily and 81.13 ± 53.22 for children receiving 1000 IU daily, with a *p*-value of 0.9 (Table 2 and Figure 2 and Figure 3). The change in 25(OH)D concentration from randomization till 6 months was −25.72 ± 31.83 in the 400 IU group and −27.15 + 65.6 in the 1000 IU group (*p*-value 0.94). Table 3 shows the univariate regression analysis of the impact of taking enzyme inducer therapy on a child‘s BMI on 25(OH)D concentration. Only obesity seems to have a negative effect on 25(OH)D concentration, but it was not statistically significant.

The treatment failure frequency was similar between the groups at randomization and 6 months post-treatment. There were no patients who had total calcium > 2.7 mg/dL, 25 OH vit D concentration > 250 nmol/L, or urine calcium: creatinine ratio > 1.2 mol/mol. Moreover, the PTH level was consistently within normal across all participants, regardless of their 25 OH vitamin D concentration. The person correlation was −0.35, *p*-value < 0.001.

## 4. Discussion

Vitamin D deficiency remains prevalent worldwide. Our RCT assessed the effectiveness of using vitamin D 400 IU versus 1000 IU as a maintenance dose for children taking ASMs.

Our study found that while vitamin D levels (25(OH)D concentration) declined over time in all groups, a daily dose of 1000 IU was more effective than 400 IU in maintaining 25(OH)D concentration > 75 nmol/L, particularly in children receiving monotherapy for their epilepsy. In this group, supplementation with 1000 IU of vitamin D was needed for four children to prevent a decline of 25(OH)D concentration below 75 nmol/L. This is supported by previous literature, indicating that ASMs are associated with lower 25(OH)D concentrations, and thus, children will need higher vitamin D maintenance doses [10,11,12,13,14,15,16,17,18,19,20,21,22]. Two previous trials in children with epilepsy showed that children who were randomized to a placebo experienced a decline in 25(OH)D concentration at the end of the study compared to sustained 25(OH)D concentration in the intervention group [32,33].

However, there was no similar benefit observed among children receiving polytherapy. This may be due to our tested doses possibly not being sufficient to maintain 25(OH)D concentrations, either because they are taking more than one ASM that negatively impacts 25(OH)D concentrations and the interaction between ASMs or due to the small sample size in the polytherapy group, making it challenging to detect statistical significance. While recommending 1000 IU daily maintenance for children taking polytherapy may not fully sustain their levels, it is likely a better option than 400 IU daily, given the significant decline in levels with 400 IU. However, this remains to be further confirmed in future trials testing doses higher than 1000 IU daily.

Our study consistently observed normal PTH levels across most participants, regardless of their 25(OH)D concentration. This finding was present at baseline, during treatment for vitamin D deficiency, and at the end of the trial. The correlation between PTH and 25(OH)D concentration was weak. These findings suggest that PTH may not be an optimal biomarker for assessing vitamin D status in this population. Future research should investigate alternative biomarkers that better reflect vitamin D sufficiency and the targeted outcome.

Interestingly, during our trial, we observed that only a third of enrolled children had 25(OH)D concentration > 75 nmol/L, and almost 50% of the cohort required multiple treatment courses to improve the 25(OH)D concentration > 75 nmol/L. Moreover, the 25(OH)D concentration steadily declined during the trial while they were on maintenance therapy. The impact of vitamin D supplementation appeared independent of the child’s weight status (obesity or non-obesity) and enzyme inducer use. This further supports the notion that those children’s requirements for maintenance and treatment are greater than what is typically anticipated in clinical settings. This may be related to the effect of ASMs or genetic predisposition. The hepatic *CYP27A1* and *CYP2R1* enzymes are involved in 25-hydroxylation of ergocalciferol (D2) and cholecalciferol (D3) to 25(OH)vitD. Mutations in the *CYP27A1* gene can lead to genetic vitamin D deficiency, which typically responds poorly to the usual vitamin D deficiency treatment. Two *CYP2R1* mutation-related vitamin D deficiencies, namely c.367 + 1 G > A and c.768dupT, were reported in a cohort of 27 patients in Saudi Arabia, where there is a high rate of consanguinity [34]. In addition to single nucleotide polymorphisms (SNPs) in the group-specific component (GC), which encodes vitamin D-binding protein that transports vitamin D metabolites to target tissues [35]. The SNP polymorphism was reported to affect 25(OH)D concentration in a cohort of 244 Malaysian children with epilepsy. Therefore, future studies are needed to aid in genetic risk stratification of children with epilepsy and low 25(OH)D concentration.

Increasing 25(OH)D concentration above 75 nmol/L led to an increased number of seizure-free children. In our trial, for every 2.6 children who received a treatment course at a screening visit, one child became seizure-free before randomization to maintenance therapy. However, vitamin D supplementation does not seem to have an additional effect once the 25(OH)D concentration has increased beyond 75 nmol/L. This effect was not mediated by normalizing low calcium concentration secondary to low 25(OH)D concentration. Similarly, a pilot study in adults showed a significant 40% seizure reduction post-correction of vitamin D deficiency [24]. It is proposed that a low 25(OH)D concentration tends to increase seizure frequency in children with epilepsy. Prior research has documented a notable seasonal variation in seizure frequency, with a decrease in seizure occurrence during the summer months and a peak in winter [4,5,6]. The increased seizure frequency during winter was attributed to a low 25(OH)D concentration. This highlights that vitamin D plays a crucial role in neurotransmission and immune response in the CNS [2]. Moreover, the effects of vitamin D extend beyond bone and muscle strength to include immune function, cell proliferation, and differentiation. The expected costs incurred from such a supplement, considering its benefits, are likely favorable to parents compared to changes in the ASM type or dose, taking into consideration the possible incurred costs and expected side effects.

Our study has some limitations. First, due to early termination during the COVID-19 pandemic and challenges in recruitment, the sample size was insufficient to detect variations between subgroups. Additionally, most children exhibited vitamin D deficiency that was resistant to treatment. Second, we did not evaluate factors that could influence vitamin D levels. This includes not assessing the impact of seasonal variations on the measured 25(OH)D concentration and sunlight exposure and not evaluating dietary vitamin D intake. While no physical signs of rickets were observed in the participants, we did not perform skeletal X-rays to assess for radiological signs of rickets.

Based on the current understanding, pediatricians and neurologists should consider supplementing children with epilepsy with at least 1000 IU of vitamin D daily to maintain 25(OH)D concentration above 75 nmol/L, which may potentially improve seizure control. For children with a 25(OH)D concentration below 75 nmol/L and uncontrolled seizures, testing and treatment of vitamin D deficiency are recommended to potentially improve seizure control. Further studies are warranted to investigate the efficacy of higher vitamin D doses in children with epilepsy, use a larger sample size to test for dose–response relationships, and explore the possibility of using genetic risk stratification to guide personalized treatment approaches.

## Figures and Tables

**Table 1:** Baseline characteristics.

	Values, N (%)					
	Monotherapy			Polytherapy		
Characteristics	400 IU N = 48	1000 IU N = 42	*p*-Value	400 IU N = 11	1000 IU N = 14	*p*-Value
Mean age, years (SD)	9.08 ± 2.89	9.10 ± 3.19	0.99	8.91 ± 3.78	7.29 ± 3.87	0.30
Age groups						
<6 years	9 (18.75)	8 (19.05)	0.77	3 (27.27)	8 (57.14)	0.32
6–10 years	17 (35.42)	12 (28.57)		3 (27.27)	2 (14.29)	
>10 years	22 (45.83)	22 (52.38)		5 (45.45)	4 (28.57)	
Sex, male	26 (54.17)	21 (50.00)	0.69	5 (45.45)	6 (42.86)	0.90
Living in an urban area	43 (89.58)	38 (90.48)	0.89	10 (90.91)	13 (92.86)	0.86
Maternal education						
Illiterate	2 (4.17)	3 (7.14)	0.07	0	0	0.43
Elementary	0 (0.00)	4 (9.52)		1 (9.09)	3 (21.43)	
Intermediate	9 (18.75)	2 (4.76)		1 (9.09)	1 (7.14)	
Secondary	14 (29.17)	8 (19.05)		4 (36.36)	1 (7.14)	
Diploma	1 (2.08)	2 (4.76)		1 (9.09)	1 (7.14)	
University	22 (45.83)	23 (54.76)		4 (36.36)	8 (57.14)	
Paternal education						
Illiterate	1 (2.08)	0 (0.00)	0.07	0	0	0.70
Elementary	0 (0.00)	4 (9.52)		1 (9.09)	0 (0)	
Intermediate	3 (6.25)	8 (19.05)		1 (9.09)	2 (14.29)	
Secondary	13 (27.08)	6 (14.29)		3 (27.27)	4 (28.57)	
Diploma	4 (8.33)	3 (7.14)		0	0	
University	27 (56.25)	21 (50.00)		6 (54.55)	8 (57.14)	
Mean Height, cm (SD)	131.1 ± 16.92	130.03 ± 20.56	0.79	127.52 ± 19.24	117.51 ± 22.51	0.25
Mean Height SDS (SD)	−0.16 ± 1.12	−0.31 ± 1.10	0.51	−0.50 ± 1.33	−0.63 ± 1.58	0.830
Mean Weight, kg (SD)	33.86 ± 14.56	33.24 ± 19.57	0.86	25.79 ± 10.52	24.28 ± 12.42	0.75
Mean BMI, kg/m^2^ (SD)	18.78 ± 4.70	18.14 ± 5.53	0.56	15.39 ± 3.49	16.41 ± 3.61	0.48
Mean BMI SDS (SD)	0.26 ± 1.59	0.65 ± 6.54	0.69	−1.21 ± 2.01	−1.25 ± 4.19	0.98
Obesity	16 (33.33)	16 (38.10)	0.64	2 (18.18)	3 (21.43)	0.84
Preterm pregnancy	3 (6.38)	4 (9.52)	0.58	0 (0)	3 (21.43)	0.10
Developmental delay	13 (27.08)	16 (38.10)	0.27	6 (54.55)	8 (57.14)	0.90
Seizure type						
Generalized	39 (81.25)	28 (66.67)	0.13	5 (45.45)	7 (50)	0.52
Unknown	1 (2.08)	0 (0.00)		1 (9.09)	0 (0)	
Focal	8 (16.67)	14 (33.33)		5 (45.45)	7 (50)	
Treatment failure	11 (22.92)	14 (33.33)	0.27	8 (72.73)	11 (78.57)	0.73
Seizure frequency						
Daily	2 (4.17)	1 (2.38)		1 (9.09)	2 (14.29)	
Weekly	1 (2.08)	4 (9.52)		4 (36.36)	1 (7.14)	
Monthly	7 (14.58)	5 (11.90)		2 (18.18)	7 (50)	
6 months	1 (2.08)	4 (9.52)		1 (9.09)	1 (7.14)	
Medications						
Carbamazepine	12 (25)	16 (38.10)		4 (36.36)	4 (28.57)	
Oxcarbazepine	2 (4.17)	0		0	0	
Valproic acid	13 (27.08)	5 (11.90)		8 (72.73)	8 (57.14)	
Lamotrigine	2 (4.17)	2 (4.76)		3 (27.27)	2 (14.29)	
Levetiracetam	16 (33.33)	14 (33.33)		7 (63.64)	9 (64.29)	
Phenobarbitone	0	0		1 (9.09)	1 (7.14)	
Topiramate	2 (4.17)	1 (2.38)		2 (18.18)	6 (42.86)	
Ethosuximide	1 (2.08)	4 (9.52)		0	0	
Clonazepam	0	0		1 (9.09)	3 (21.42)	
Vigabatrin	0	0		0	1 (7.14)	
ASM level						
Normal	16 (33.33)	12 (28.57)	0.78	5 (45.45)	9 (64.29)	0.23
Low	2 (4.17)	3 (7.14)		2 (18.18)	0	
High	1 (2.08)	2 (4.76)		1 (9.09))	1 (7.14)	
not applicable	8 (16.67)	6 (14.29)		0	0	
Other medical diagnosis						
Asthma	2 (4.17)	1 (2.38)		0	2 (14.29)	
Hypothyroidism	2 (4.17)	1 (2.38)		0	2 (14.29)	
Growth hormone deficiency	0	0		0	0	
ADHD	5 (10.42)	5 (11.90)		1 (9.09)	1 (7.14)	
Autistic spectrum disorder	1 (2.08)	1 (2.38)		0	0	
Spastic quadriplegia CP	0	3 (7.14)		2 (18.18)	0	
Brain malformation	3 (6.25)	2 (4.76)		2 (18.18)	0	
Others	7 (14.58)	3 (7.14)		3 (27.27)	4 (28.57)	
Concomitant medications						
Pyridoxin	2 (4.17)	1 (2.38)		0	2 (14.29)	
Risperidone	1 (2.08)	3 (7.14)		2 (18.18)	0	
Methylphenidate	1 (2.08)	2 (4.76)		1 (9.09)	0	
Baclofen	0	3 (7.14)		0	0	
Family history of vitamin D deficiency	15 (31.25)	7 (16.67)	0.10	2 (18.18)	4 (28.57)	0.55
Previous treatment with vitamin D	29 (60.42)	17 (40.48)	0.06	2 (18.18)	7 (50)	0.10
Mean parent-reported sun exposure, h/day (SD)	0.62 ± 1.20	0.55 ± 0.76	0.74	0.90 ± 0.77	0.08 ± 0.19	0.0013
Mean Outdoor time, hr/day (SD)	3.86 ± 2.67	2.97 ± 2.66	0.13	3.00 ± 2.68	3.15 ± 3.08	0.90
Mean Screen time, hr/day (SD)	2.89 ± 2.58	2.61 ± 2.17	0.59	1.95 ± 2.55	2.68 ± 2.46	0.48
Mean Physical activity time, h/day (SD)	1.26 ± 1.56	0.86 ± 1.18	0.18	1.86 ± 2.20	1.84 ± 2.18	0.98
Mean 25(OH)D concentration, nmol/L	66.98 ± 30.61	60.80 ± 27.49	0.31	74.90 ± 38.4	72.07 ± 52.84	0.88
25(OH)D concentration < 50 nmol/L	15 (31.25)	20 (47.62)	0.11	3 (27.27)	6 (42.86)	0.42
25(OH)D concentration < 75 nmol/L	30 (62.50)	31 (73.81)	0.25	7 (63.64)	9 (64.29)	0.97
Mean total calcium, mmol/L (SD)	2.35 ± 0.10	2.36 ± 0.12	0.84	2.31 ± 0.068	2.33 ± 0.10	0.62
Mean phosphorus, mmol/L (SD)	1.49 ± 0.19	1.46 ± 0.19	0.58	1.52 ± 0.25	1.51 ± 0.24	0.93
Mean PTH (SD), Pmol/L	4.50 ± 2.04	4.59 ± 3.79	0.86	3.51 ± 1.63	6.30 ± 5.65	0.13
Mean AST, (IU/L) (SD)	24.02 ± 5.41	26.83 ± 12.63	0.18	27.7 ± 11.05	29.69 ± 10.74	0.67
Mean ALT, (IU/L) (SD)	21.31 ± 6.84	22.37 ± 20.43	0.46	24 ± 8.14	20.15 ± 8.14	0.30
Mean GGT, (IU/L) (SD)	20.11 ± 11.05	27.68 ± 23.59	0.06	28.4 ± 20.85	34.92 ± 33.55	0.60
Mean Urine calcium to creatinine ratio (SD)	0.42 ± 1.02	0.25 ± 0.23	0.35	0.24 ± 0.30	0.22 + 0.23	0.91

PTH, parathyroid hormone.

**Table 2:** Outcomes of vitamin D supplementation.

	Values, mean ± SD											
	Monotherapy						Polytherapy					
Outcome Measure	3 Months N = 90			6 Months N = 90			3 Months N= 25			6 Months N = 25		
	400 IU	1000 IU	*p*-Value	400 IU	1000 IU	*p*-Value	400 IU	1000 IU	*p*-Value	400 IU	1000 IU	*p*-Value
No.	48	42		48	42		11	14		11	14	
25(OH)D concentration ITT, nmol/L	76.59 ± 28.65	86.73 ± 28.44	0.10	61.35 ± 22.45	73.09 ± 29.03	0.03	67.47 ± 25.77	86.83 ± 27.43	0.09	78.79 ± 23.33	81.13 ± 53.22	0.89
25(OH)D concentration < 50 nmol/L ITT, N (%)	11 (22.92)	14 (33.33)	0.01	17 (35.42)	11 (26.19)	0.35	4 (36.36)	2 (14.29)	0.19	0	4 (28.57)	0.05
25(OH)D concentration < 75 nmol/L ITT, N (%)	24 (50)	15 (35.71)	0.15	36 (75.00)	23 (54.76)	0.04	6 (54.55)	4 (28.57)	0.18	5 (45.45)	9 (64.29)	0.35
Total Calcium, mmol/L	2.34 ± 0.06	2.31 ± 0.11	0.33	2.34 ± 0.08	2.29 ± 0.94	0.04	2.34 ± 0.08	2.34 ± 0.10	0.10	2.27 ± 0.11	2.35 ± 0.13	0.22
Phosphorus, mmol/L	-	-	-	1.54 ± 0.21	1.48 ± 0.19	0.28	-	-	-	1.49 ± 0.22	1.53 ± 0.26	0.75
PTH, Pmol/L	5.05 ± 2.65	4.43 ± 2.42	0.38	5.34 ± 3.87	4.24 ± 2.65	0.17	3.51 ± 0.63	4.04 ± 3.62	0.71	3.85 ± 1.39	3.84 ± 2.88	0.99
Urine calcium to creatinine ratio	0.29 ± 0.60	0.36 ± 0.45	0.64	0.29 ± 0.64	0.20 ± 0.20	0.48	0.22 ± 0.24	0.24 ± 0.28	0.9	0.25 ± 0.29	0.19 ± 0.31	0.68
Composite outcome treatment failure ITT, N (%)	-	-	-	12 (25.00)	13 (30.95)	0.53	-	-	-	5 (45.45)	8 (57.14)	0.90
New ASM added, N (%)	-	-	-	1 (2.33)	3 (8.11)	0.24	-	-	-	1 (9.09)	0	0.23
Current ASM dose increased, N (%)	7 (14.89)	10 (24.39)	0.26	7 (16.28)	3 (8.11)	0.27	-	-	-	2 (18.18)	4 (28.57)	0.63
Seizure frequency, N (%)												
Seizure-free	-	-	-	41 (85.41)	33 (78.50)	0.37	-	-	-	5 (45.45)	9 (64.29)	0.48
Daily	-	-	-	1 (2.08)	1 (2.38)		-	-	-	1 (9.09)	2 (14.29)	
Weekly	-	-	-	1 (2.08)	3 (7.14)		-	-	-	3 (27.27)	0	
Monthly	-	-	-	4 (8.33)	3 (7.14)		-	-	-	1 (9.09)	2 (14.29)	
6 months	-	-	-	1 (2.08)	2 (4.76)		-	-	-	1 (9.09)	1 (7.14)	

ITT, intention to treat analysis.

**Table 3:** Univariate regression analysis of the impact of participant characteristics on 25(OH)D concentration post 6 months of receiving supplementation.

	Monotherapy			Polytherapy		
	B-Coefficient	95%CI	*p*-Value	B-Coefficient	95%CI	*p*-Value
Vitamin D dose 1000 IU	11.75	0.94, 22.55	0.033	2.34	−33.39, 38.07	0.89
Enzyme inducer	2.45	−9.37,14.28	0.68	−23.76	−57.76, 10.25	0.16
Obesity	−7.22	−18.67, 4.23	0.21	−0.18	−44.54, 44.18	0.99

## Data Availability

The data that support the findings of this study are available upon reasonable request from the corresponding author due to required ethical approval for datahsaring.
